# Assessing Response and Outcomes to Modern Multiagent Neoadjuvant Therapy for Pancreatic Ductal Adenocarcinoma

**DOI:** 10.1245/s10434-026-19990-7

**Published:** 2026-06-19

**Authors:** Paul H. McClelland, Megan A. Satyadi, Ryan P. Johnson, Rosetta Jacob, Jianmin Pan, Jayesh Rai, Shesh Rai, Davendra Sohal, Olugbenga Olowokure, Jordan Kharofa, Gregory C. Wilson, Sameer H. Patel, Syed A. Ahmad

**Affiliations:** 1 Department of Surgery, University of Cincinnati Medical Center, Cincinnati, OH USA; 2 Biostatistics and Informatics Shared Resource, University of Cincinnati Cancer Center, University of Cincinnati, Cincinnati, OH USA; 3 Department of Internal Medicine, Division of Hematology and Oncology, University of Cincinnati Medical Center, Cincinnati, OH USA; 4 Department of Radiation Oncology, University of Cincinnati Medical Center, Cincinnati, OH USA; 5 Division of Surgical Oncology, University of Cincinnati Medical Center, Cincinnati, OH USA

## Abstract

**Background:**

Neoadjuvant therapy (NAT) is increasingly used for resectable and borderline-resectable pancreatic ductal adenocarcinoma (PDAC), yet interpretation of treatment response remains challenging.

**Patients and Methods:**

A single-center retrospective review was conducted of patients with resectable or borderline-resectable PDAC treated with multiagent NAT between 2008 and 2024. Radiographic response was assessed using RECIST 1.1 criteria, biochemical response by serum CA 19-9 reduction, and histopathologic response by College of American Pathologists (CAP) score. Primary and secondary outcomes were overall survival (OS) and progression-free survival (PFS).

**Results:**

Among 166 patients, 148 (89%) completed NAT and 121 (73%) underwent resection. After NAT, radiographic response was uncommon: 27% had tumor regression, 66% stable disease, and 7% progressive disease. By contrast, 83% achieved ≥ 45% CA 19-9 reduction after NAT, and 74% demonstrated histopathologic response after resection, including 8% complete pathologic response. Among resected patients with radiographically stable/progressive disease, 88% had biochemical response and 68% at least partial histopathologic response. Neither radiographic nor biochemical response were significantly associated with improved PFS or OS. Histopathologic response (CAP 0–2) was associated with improved PFS/OS on univariable analysis as well as improved PFS on multivariable analysis [adjusted hazard ratio (aHR) 0.57, 95% confidence interval (CI) 0.34–0.96; *p* = 0.034]. Isolated unresectable local progression during NAT was rare (*n* = 2).

**Conclusions:**

While imaging response and downstaging can occur with NAT, histopathologic response is more predictive of long-term survival and often occurs despite stable or progressive disease on imaging. Isolated unresectable local progression after NAT is rare, though broader treatment-related attrition remains a consideration in neoadjuvant sequencing.

**Supplementary Information:**

The online version contains supplementary material available at 10.1245/s10434-026-19990-7.

Despite advances in treatment, pancreatic ductal adenocarcinoma (PDAC) remains one of the most aggressive solid malignancies and a leading cause of cancer-related mortality, with a 5-year overall survival rate of approximately 13%.^1–3^ Surgical resection is the only curative treatment; however, even among patients with localized disease, long-term survival remains limited by early recurrence and occult metastatic spread.^4–6^ In response to this trend, the role of neoadjuvant therapy (NAT) in PDAC has expanded in recent years, with many patients with resectable and borderline-resectable disease undergoing modern multiagent chemotherapy regimens with or without radiation prior to resection.^3,4^ The goals of this treatment strategy are several-fold: early treatment of occult metastatic disease, testing tumor biology, improving patient selection for surgery, and downstaging locally advanced tumors.^3–5,7^ To date, contemporary regimens with folinic acid, 5-flurouracil, irinotecan, and oxaliplatin (FOLFIRINOX) and gemcitabine/nab-paclitaxel have been studied in numerous clinical trials and have demonstrated survival benefit, decreased recurrence, and higher rates of achieving margin-negative (R0) resection over historical single-agent therapy.^7–11^

While NAT has become increasingly widespread in the management of PDAC, assessment of response to therapy remains challenging and has important implications for surgical decision-making. Treatment sequencing in resectable and borderline-resectable PDAC continues to generate debate, driven by concern that prolonged neoadjuvant therapy may result in loss of the therapeutic window for potentially curative resection.^12,13^ Additionally, the optimal interpretation of poor or absent response to NAT remains uncertain, which may contribute to hesitation in progression to surgery in patients without clear evidence of treatment response. Radiographic and biochemical response are frequently used to assess treatment effect after NAT; however, their prognostic relevance and concordance with oncologic outcomes remain incompletely defined. Radiographic response is most commonly determined by Response Evaluation Criteria in Solid Tumors (RECIST), however several studies have demonstrated that RECIST may overestimate tumor burden and underestimate true response to treatment as assessed on final histopathologic response.^14–16^ Similarly, biochemical response, most commonly assessed by reduction in serum carbohydrate antigen 19-9 (CA 19-9), has been shown to correlate with treatment effect and oncologic outcomes, but the optimal degree of decline and whether normalization is required to confer prognostic benefit has yet to be determined.^17–21^ Histopathologic response provides an additional measure of treatment effect following NAT, but its assessment remains heterogeneous owing to multiple tumor regression grading systems with variable definitions, limited concordance, and inconsistent associations with oncologic outcomes.^22,23^ Moreover, studies suggest that quantitative assessment of residual viable tumor by the College of American Pathologists (CAP) scoring system may better reflect treatment response and prognosis than categorical tumor regression grading alone.^24^

Given the uncertainty surrounding interpretation of radiographic, biochemical, and histopathologic response after neoadjuvant therapy, this study aims to systematically evaluate these response domains in a contemporary cohort of patients with resectable and borderline-resectable PDAC who underwent modern multiagent NAT. Additionally, this analysis evaluates whether poor or absent response to NAT is associated with inferior oncologic outcomes, with the goal of informing surgical decision-making in the neoadjuvant setting.

## Patients and Methods

### Study Site and Patient Enrollment

This study is a single-center retrospective review of a prospectively collected database maintained at an urban, tertiary-level university hospital between 2008 and 2024. Data were obtained from an ongoing registry of patients undergoing treatment for PDAC via a combination of surgery and systemic therapy, with documentation of study variables from time of diagnosis through treatment, as well as longitudinal follow-up. Patients in this database were primarily enrolled for participation in randomized controlled trials and therefore underwent standardized workup, treatment, and follow-up according to protocol. Details regarding these standardized protocols have been previously published elsewhere.^25^

### Analysis Design, Variables, and Outcomes

The primary inclusion criteria for this study were patients aged 18–89 years receiving NAT for PDAC. Patients included in this study followed a standard treatment course including initial outpatient workup, followed by one of two standardized neoadjuvant chemotherapy regimens: modified FOLFIRINOX or gemcitabine/nab-paclitaxel. Completion of NAT was defined as successful delivery of all assigned treatments per protocol, as well as subsequent recovery from these treatments. After neoadjuvant therapy, select patients underwent radiation therapy, and most underwent resection (pancreatoduodenectomy *vs.* distal pancreatectomy), followed by longitudinal postoperative monitoring.

Variables including patient demographics, baseline characteristics, tumor attributes, treatment categories, intraoperative factors, and survival were collected throughout each patient’s clinical course. Eastern Cooperative Oncology Group (ECOG) scores were used to assess baseline functional status, National Cancer Institute (NCI) comorbidity indices were used to assess comorbidities, and National Comprehensive Cancer Network (NCCN) Resectability Classifications were used to distinguish between resectable, borderline-resectable, and locally advanced tumors. Three response metrics were evaluated: radiologic tumor response as measured by RECIST 1.1 criteria, biochemical response as measured by serum CA 19-9 reduction, and histopathologic response as measured by College of American Pathologists (CAP) score. CA 19-9 reduction thresholds were adopted from prior work performed to determine CA 19-9 cutoffs in a similar modern cohort, which found that ≥ 45% decline was associated with improved survival with ≥ 85% being the optimal threshold.^19^ Moreover, using a standard normal threshold of ≤ 37 U/mL, CA 19-9 nonproducers were defined as patients whose serum CA 19-9 levels were within normal limits both before and after neoadjuvant therapy; these patients were excluded from CA 19-9 calculations. The primary and secondary outcomes were overall survival (OS) and progression-free survival (PFS), measured from date of diagnosis.

### Statistical Analysis and Ethics

Descriptive statistics were used to summarize preoperative patient characteristics, treatment-related factors, and postoperative outcomes. Continuous variables were reported as medians with interquartile ranges [Q1–Q3], which were compared via Mann-Whitney *U* test. Categorical variables were expressed as proportions and compared using *χ*^2^ tests. Survival outcomes were reported by median survival and compared with log-rank *p*, and all descriptive tests were two-tailed with *p* ≤ 0.05 considered significant. For predictive analyses, regression analysis was performed using first a univariable screen for significant predictors (threshold *p* ≤ 0.05), followed by multivariable Cox proportional hazard modeling using PFS and OS as endpoints. The R statistical programming language (v4.5.2, R Foundation for Statistical Computing, Vienna, Austria) was used for all statistical calculations.

All patient data were de-identified and encrypted prior to inclusion in the digital registry, which was accessed only by dedicated research staff. Ethical clearance for this study was obtained by institutional review board (IRB).

## Results

### Overall Cohort and Baseline Characteristics

In total, 166 patients were included in the overall cohort. Of these, 53% were male, 86% were white/Caucasian, and the median age was 66 [interquartile range (IQR) 60–73] years (Table 1). In general, included patients had good functional status (ECOG 0 64%) and few comorbidities (median NCI Comorbidity Index 0.3 [0.0–0.4]). T2 tumors were most prevalent (48%), with an average tumor diameter of 2.9 [2.3–3.6] cm. At baseline, 58% of patients were resectable, and 42% of patients were borderline-resectable. Most patients were clinically stage N0 at time of diagnosis (67%), and the median baseline serum CA 19-9 level was 293 [136–798] U/mL.

**Table 1:** Demographics and disease characteristics of the overall cohort

Variable	Total (*N* = 166)
Age, years, median [IQR]	66.2 [60.1–73.3]
Sex	–
Female	78 (47.0%)
Male	88 (53.0%)
Race/ethnicity	–
White or Caucasian	143 (86.1%)
Black or African American	23 (13.9%)
ECOG Performance Status	–
0	96 (64.4%)
1	53 (35.6%)
NCI Comorbidity Index, median [IQR]	0.3 [0.0–0.4]
Tumor size (largest dimension), median [IQR]	2.9 [2.3–3.6]
Clinical T stage	–
T1	27 (18.0%)
T2	72 (48.0%)
T3	16 (10.7%)
T4	35 (23.3%)
Clinical N stage	–
N0	101 (67.3%)
N1-2	49 (32.7%)
NCCN Resectability Classification, baseline	–
Resectable	96 (57.8%)
Borderline-resectable	70 (42.2%)
Baseline serum CA 19-9 level (U/mL), median [IQR]	292.9 [135.6–798.0]

*IQR* interquartile range, *ECOG* Eastern Cooperative Oncology Group, *NCI* National Cancer Institute

### Neoadjuvant Therapy and Preoperative Factors

Of 166 patients, 148 (89%) were able to complete NAT (Supplementary Fig. 1). Among the 18 patients unable to complete NAT, 12 were unable to tolerate therapy (6 converted to palliative treatment, 4 died during treatment, and 2 had expedited resection) and 6 were lost to follow-up. Of the 148 patients able to complete neoadjuvant therapy, 121 (82%) were ultimately able to undergo resection, yielding an overall resection rate of 73%. Among the 27 patients forgoing resection, 2 (7%) had unacceptable comorbidities risk, 2 (7%) declined surgery, and 23 (66%) had progressive disease; among those with progressive disease, 11 had occult intraperitoneal disease and 10 had distant extraperitoneal metastasis, with only 2 patients demonstrating isolated unresectable local progression on NAT preventing surgical intervention. These outcomes were broadly consistent with those found on imaging, with comparatively higher rates of post-neoadjuvant dropout seen among patients with stable disease (SD 24%) and progressive disease (PD 27%) by radiographic criteria compared to those with evidence of complete/partial disease response on imaging (3%).

The two patients who experienced isolated unresectable local progression on NAT had a similar presentation and disease course. The first patient presented with borderline-resectable disease with > 180° involvement of the superior mesenteric vein (SMV). The patient completed seven cycles of neoadjuvant modified FOLFIRINOX, requiring dose reductions owing to treatment-related intolerance. At 4 months after initiation of NAT, interval CT imaging demonstrated disease progression with > 180° encasement of the common hepatic and gastroduodenal arteries, and the patient was transitioned to gemcitabine/nab-paclitaxel. The second patient presented with borderline-resectable disease owing to < 180° involvement of the superior mesenteric artery (SMA) and < 90° involvement of the SMV. The patient completed eight cycles of modified FOLFIRINOX with dose reductions owing to treatment-related toxicity. Interval imaging four months after starting NAT demonstrated progression with increasing SMA encasement and new involvement of the first jejunal vein, prompting transition to gemcitabine/nab-paclitaxel.

Patients undergoing resection were not substantially different from the overall cohort in terms of demographics and baseline disease characteristics (Table 2). Approximately half of these patients underwent gemcitabine-based chemotherapy (54%), with the other half receiving FOLFIRINOX-based chemotherapy (46%). Most of these patients received radiation therapy (69%), followed by pancreatoduodenectomy (83%) with negative margins (88%). Classical prognostic indicators, such as tumor grade and lymph node status, were not significantly different between patients with and without imaging response, although patients with observed radiographic response were more frequently found to have higher T stage at baseline (T3–4 46% *vs.* 26%; *p* = 0.040).

**Table 2:** Perioperative factors in patients with resectable and borderline-resectable PDAC undergoing neoadjuvant therapy and resection, stratified by imaging response criteria

Variable	Total (*N* = 121)	No response (SD + PD) (*N* = 82)	Response (CR + PR) (*N* = 39)	*p*-Value
Age, years, median [IQR]	65.9 [60.1–72.7]	65.7 [59.3–72.7]	67.8 [63.0–73.2]	0.197
Sex	–	–	–	0.546
Female	56 (46.3%)	40 (48.8%)	16 (41.0%)	–
Male	65 (53.7%)	42 (51.2%)	23 (59.0%)	–
Race/ethnicity	–	–	–	1.000
White or Caucasian	106 (87.6%)	72 (87.8%)	34 (87.2%)	–
Black or African American	15 (12.4%)	10 (12.2%)	5 (12.8%)	–
ECOG performance status	–	–	–	0.597
0	80 (66.1%)	56 (68.3%)	24 (61.5%)	–
1	41 (33.9%)	26 (31.7%)	15 (38.5%)	–
NCI Comorbidity Index, median [IQR]	0.3 [0.0–0.4]	0.3 [0.0–0.4]	0.0 [0.0–0.3]	0.597
Tumor size (largest dimension), median [IQR]	2.9 [2.2–3.6]	2.8 [2.0–3.5]	3.0 [2.5–3.8]	0.077
Clinical T stage	–	–	–	**0.040**
T1	24 (19.8%)	19 (23.2%)	5 (12.8%)	–
T2	58 (47.9%)	42 (51.2%)	16 (41.0%)	–
T3	14 (11.6%)	5 (6.1%)	9 (23.1%)	–
T4	25 (20.7%)	16 (19.5%)	9 (23.1%)	–
Clinical N stage	–	–	–	0.907
N0	80 (66.1%)	55 (67.1%)	25 (64.1%)	–
N1-2	41 (33.9%)	27 (32.9%)	14 (35.9%)	–
NCCN Resectability Classification, baseline	–	–	–	1.000
Resectable	70 (57.9%)	47 (57.3%)	23 (59.0%)	–
Borderline-resectable	51 (42.1%)	35 (42.7%)	16 (41.0%)	–
Baseline serum CA 19-9 level (U/mL), median [IQR]	270.0 [130.8–725.0]	319.0 [135.7–906.0]	186.0 [96.3–477.4]	0.118
Neoadjuvant chemotherapy regimen	–	–	–	0.861
FOLFIRINOX-based	56 (46.3%)	37 (45.1%)	19 (48.7%)	–
Gemcitabine-based	65 (53.7%)	45 (54.9%)	20 (51.3%)	–
Neoadjuvant radiation	–	–	–	0.916
No	38 (31.4%)	25 (30.5%)	13 (33.3%)	–
Yes	83 (68.6%)	57 (69.5%)	26 (66.7%)	-
Duration of neoadjuvant therapy, months, median [IQR]	4.0 [3.2–5.0]	4.0 [2.9–5.0]	4.1 [3.3–5.0]	0.333
Post-treatment resectable status change (simplified)	–	–	–	0.261
Unchanged	96 (79.3%)	65 (79.3%)	31 (79.5%)	–
Downstage	20 (16.5%)	12 (14.6%)	8 (20.5%)	–
Upstage	5 (4.1%)	5 (6.1%)	0 (0.0%)	–
Post-treatment serum CA 19-9 level (U/mL), median [IQR]	48.8 [28.8–86.0]	51.3 [30.4–128.9]	40.0 [22.6–65.9]	0.088
Post-neoadjuvant serum CA 19-9 reduction from baseline	–	–	–	0.846
< 45% reduction	9 (10.7%)	7 (12.5%)	2 (7.1%)	–
45–85% reduction	41 (48.8%)	27 (48.2%)	14 (50.0%)	–
≥ 85% reduction	34 (40.5%)	22 (39.3%)	12 (42.9%)	–
Surgical resection	–	–	–	1.000
Pancreatoduodenectomy	100 (82.6%)	68 (82.9%)	32 (82.1%)	–
Distal pancreatectomy	21 (17.4%)	14 (17.1%)	7 (17.9%)	–
Positive margin on specimen	–	–	–	0.382
No	106 (87.6%)	70 (85.4%)	36 (92.3%)	–
Yes	15 (12.4%)	12 (14.6%)	3 (7.7%)	–
College of American Pathologists treatment effect	–	–	–	**0.008**
Poor or no response—Score 3	31 (25.6%)	26 (31.7%)	5 (12.8%)	–
Partial response—Score 2	65 (53.7%)	45 (54.9%)	20 (51.3%)	–
Near-complete response—Score 1	15 (12.4%)	8 (9.8%)	7 (17.9%)	–
Complete response—Score 0	10 (8.3%)	3 (3.7%)	7 (17.9%)	–
Histopathological tumor grade	–	–	–	0.323
Well-differentiated—Grade 1	19 (18.3%)	16 (21.6%)	3 (10.0%)	–
Moderately differentiated—Grade 2	53 (51.0%)	35 (47.3%)	18 (60.0%)	–
Poorly differentiated—Grade 3	32 (30.8%)	23 (31.1%)	9 (30.0%)	–
Positive lymph nodes in specimen	–	–	–	0.263
No	64 (52.9%)	40 (48.8%)	24 (61.5%)	–
Yes	57 (47.1%)	42 (51.2%)	15 (38.5%)	–
Progression-free survival, median (95% CI)	25.0 (18.0–33.4)	23.5 (17.8–38.0)	29.0 (16.2–NA)	0.210
Overall survival, median (95% CI)	35.6 (32.2–45.4)	35.6 (33.4–45.4)	30.3 (27.0–NA)	0.521

Bold text denotes significant *p* ≤ 0.05

*CR* complete response, *PR* partial response, *IQR* interquartile range, *CI* confidence interval, *ECOG* Eastern Cooperative Oncology Group, *NCI* National Cancer Institute, *NCCN* National Comprehensive Cancer Network

### Radiographic Response

Most patients who underwent resection after NAT had radiographic stable disease prior to surgery (SD 61%), with a substantial minority showing complete or partial response after NAT (CR/PR 32%). Similarly, most patients maintained their resectability status after NAT (79%), with 17% experiencing downstage in resectability criteria after neoadjvant treatment. There was a small subset of patients who underwent resection that progressed from resectable to borderline-resectable disease after neoadjuvant therapy (4%).

### Biochemical Response

Of 121 patients who underwent resection, 16 (13%) had incomplete CA 19-9 data (missing either baseline or post-neoadjuvant values) and 21 (17%) were nonproducers, leaving 84 (70%) evaluable for biochemical response analysis. Among this cohort, most exhibited some degree of post-neoadjuvant biochemical response, with 89% of patients achieving ≥ 45% reduction in serum CA 19-9 from baseline, and 41% of patients achieving ≥ 85% reduction in CA 19-9. The frequency of CA 19-9 reduction was similar between patients with and without imaging response. Examining only the individuals with stable disease by RECIST, 46 of 74 (62%) had a confirmed ≥ 45% decrease in CA 19-9 following neoadjuvant therapy.

### Histopathologic Response

By CAP score criteria, most patients were observed to have some degree of histopathological response after resection, with 54% demonstrating partial response (CAP 2) and 21% demonstrating complete or near-complete response (CAP 0-1). When stratifying patients by imaging response criteria, patients with poor imaging response (SD/PD) were found to have a higher proportion of unfavorable CAP scores on histopathology (CAP 3 32% *vs.* 13%; *p* = 0.008); however, most of these patients still had favorable histopathological response (68%) despite having an unfavorable imaging response (Table 2).

### Prognostic Indicators and Survival

**Fig. 1:**
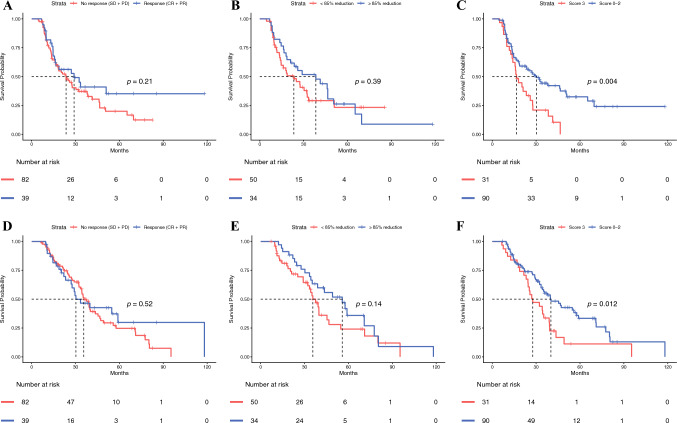
Kaplan–Meier survival curves showcasing progression-free survival (PFS) and overall survival (OS), stratified by response criteria. **A** PFS stratified by imaging criteria, **B** PFS stratified by biochemical criteria, **C** PFS stratified by histopathological criteria, **D** OS stratified by imaging criteria, **E** OS stratified by biochemical criteria, and **F** OS stratified by histopathological criteria

Post-resection analysis revealed some correlation between tumor response metrics and PFS/OS (Fig. 1). The metric with the strongest association with increased survival was histopathologic response (CAP 0–2), with improved median PFS (30 *vs.* 17 months; *p* = 0.004) and OS (40 *vs.* 28 months; *p* = 0.012). By contrast, neither response by biochemical criteria (≥ 85%) or response by imaging criteria (CR/PR) were significantly associated with improved PFS or OS (30 *vs.* 35 months; *p* = 0.41), and patients with combined absent radiographic and biochemical response did not demonstrate significantly worse PFS or OS compared with those with any measurable response (Supplementary Fig. 2). Considering NAT regimens, there was no survival benefit seen with neoadjuvant radiation or FOLFIRINOX- and gemcitabine-based chemotherapy regimens.

Similar associations were seen in multivariable analysis. Using PFS as an endpoint, CAP 0–2 was found to be independently associated with improved survival adjusted hazard ratio (aHR) 0.57, 95% confidence interval (CI) 0.34–0.96; *p* = 0.034), whereas baseline borderline/nonresectable baseline resectability criteria, disease and positive lymph nodes were found to be independently associated with worse PFS (Table 3). By contrast, while CAP 0–2 status (HR 0.55, 95% CI 0.35–0.89; *p* = 0.014) was found to be significantly associated with improved OS, this association was not upheld in multivariable analysis, in which only borderline baseline resectability and lymphatic spread were independently associated with worse survival (Table 4). In a sensitivity analysis examining CAP score as a three-level variable (Score 0-1, Score 2, Score 3), both Score 0-1 and Score 2 were associated with improved PFS and OS on univariable analysis (Supplementary Fig. 3). On multivariable analysis, Score 2 retained an independent association with survival, while the Score 0-1 effect was attenuated by collinearity with favorable pathologic features including lymph node negativity.

**Table 3:** Univariable screen and multivariable Cox regression analysis of factors affecting progression-free survival (PFS) in patients with resectable or borderline-resectable PDAC undergoing neoadjuvant therapy and resection

Variable	Group	*n*	Events	HR (95% CI)	Uni *p*	aHR (95% CI)	Multi *p*
Age, years	–	121	77	1.00 (0.98–1.03)	0.970	–	–
Sex	Female	56	37	Reference	–	–	–
	Male	65	40	0.87 (0.55–1.35)	0.528	–	–
Race/ethnicity	White or Caucasian	106	64	Reference	–	Reference	–
	Black or African American	15	13	1.82 (1.00–3.32)	0.050	1.87 (0.99–3.55)	0.055
ECOG Performance Status	0	80	50	Reference	–	–	–
	1	41	27	0.99 (0.62–1.59)	0.973	–	–
NCI Comorbidity Index	–	121	77	0.85 (0.42–1.69)	0.637	–	–
Tumor size (largest dimension)	–	121	77	1.10 (0.89–1.36)	0.372	–	–
Clinical T stage	T1	24	14	Reference	–	–	–
	T2	58	39	1.55 (0.84–2.87)	0.161	–	–
	T3	14	6	1.03 (0.40–2.70)	0.945	–	–
	T4	25	18	1.80 (0.89–3.62)	0.100	–	–
Clinical N stage	N0	80	50	Reference	–	–	–
	N1-2	41	27	0.87 (0.54–1.40)	0.560	–	–
NCCN Resectability Classification, baseline	Resectable	70	36	Reference	–	Reference	–
	Borderline-resectable	51	41	2.22 (1.42–3.49)	**< 0.001**	3.01 (1.87–4.87)	**< 0.001**
Baseline serum CA 19-9 level	–	121	77	1.00 (1.00–1.00)	0.704	–	–
Neoadjuvant chemotherapy regimen	FOLFIRINOX-based	56	33	Reference	–	–	–
	Gemcitabine-based	65	44	1.01 (0.64–1.59)	0.969	–	–
Neoadjuvant radiation	No	38	22	Reference	–	–	–
	Yes	83	55	1.01 (0.61–1.66)	0.965	–	–
Duration of neoadjuvant therapy, months	–	121	77	0.89 (0.79–1.00)	0.053	–	–
Post-treatment RECIST 1.1 score (simplified)	No response (SD + PD)	82	56	Reference	–	–	–
	Response (CR + PR)	39	21	0.73 (0.44–1.20)	0.211	–	–
Post-treatment resectable status change (simplified)	Unchanged	96	58	Reference	–	–	–
	Downstage	20	15	1.65 (0.93–2.92)	0.086	–	–
	Upstage	5	4	1.36 (0.49–3.76)	0.555	–	–
Post-neoadjuvant serum CA 19-9 reduction from baseline	< 45% reduction	9	6	Reference	–	–	–
	45–85% reduction	41	26	1.12 (0.46–2.74)	0.806	–	–
	≥ 85% reduction	34	24	0.87 (0.35–2.13)	0.757	–	–
Surgical resection	Pancreatoduodenectomy	100	61	Reference	–	–	–
	Distal pancreatectomy	21	16	1.22 (0.70–2.12)	0.482	–	–
Positive margin on specimen	No	106	66	reference	–	–	–
	Yes	15	11	1.51 (0.79–2.87)	0.208	–	–
College of American Pathologists treatment effect	Score 3	31	25	Reference	–	Reference	–
	Score 0–2	90	52	0.49 (0.30–0.80)	**0.005**	0.57 (0.34–0.96)	**0.034**
Histopathological tumor grade	Well-differentiated–Grade 1	19	11	Reference	–	–	–
	Moderately differentiated–Grade 2	53	35	1.64 (0.83–3.24)	0.154	–	–
	Poorly differentiated–Grade 3	32	23	1.70 (0.83–3.50)	0.147	–	–
Positive lymph nodes in specimen	No	64	35	Reference	–	Reference	–
	Yes	57	42	2.79 (1.74–4.46)	**< 0.001**	2.77 (1.70–4.51)	**< 0.001**

Bold text denotes significant *p* ≤ 0.05

*CI* confidence interval, *ECOG* Eastern Cooperative Oncology Group, *NCI* National Cancer Institute, *NCCN* National Comprehensive Cancer Network

**Table 4:** Univariable screen and multivariable Cox regression analysis of factors affecting overall survival (OS) in patients with resectable or borderline-resectable PDAC undergoing neoadjuvant therapy and resection

Variable	Group	*n*	Events	HR (95% CI)	Uni *p*	aHR (95% CI)	Multi *p*
Age, years	–	121	78	0.99 (0.97–1.02)	0.681	–	–
Sex	Female	56	37	Reference	–	–	–
	Male	65	41	0.89 (0.57–1.39)	0.599	–	–
Race/ethnicity	White or Caucasian	106	66	Reference	–	–	–
	Black or African American	15	12	1.41 (0.76–2.65)	0.278	–	–
ECOG Performance Status	0	80	52	Reference	–	–	–
	1	41	26	0.95 (0.59–1.53)	0.833	–	–
NCI Comorbidity Index	–	121	78	0.86 (0.43–1.70)	0.660	–	–
Tumor size (largest dimension)	–	121	78	1.13 (0.90–1.41)	0.305	–	–
Clinical T stage	T1	24	16	Reference	–	–	–
	T2	58	36	1.17 (0.65–2.12)	0.605	–	–
	T3	14	9	1.08 (0.45–2.56)	0.867	–	–
	T4	25	17	1.66 (0.83–3.32)	0.151	–	–
Clinical N stage	N0	80	51	Reference	–	–	–
	N1-2	41	27	0.71 (0.44–1.15)	0.168	–	–
NCCN Resectability Classification, baseline	Resectable	70	39	Reference	–	Reference	–
	Borderline-resectable	51	39	1.64 (1.04–2.58)	**0.033**	1.98 (1.23–3.21)	**0.005**
Baseline serum CA 19-9 level	–	121	78	1.00 (1.00–1.00)	0.726	–	–
Neoadjuvant chemotherapy regimen	FOLFIRINOX-based	56	26	Reference	–	Reference	–
	Gemcitabine-based	65	52	1.68 (1.04–2.71)	**0.033**	1.42 (0.86–2.34)	0.172
Neoadjuvant radiation	No	38	18	Reference	–	–	–
	Yes	83	60	1.06 (0.62–1.80)	0.835	–	–
Duration of neoadjuvant therapy, months	–	121	78	0.97 (0.89–1.06)	0.517	–	–
Post-treatment RECIST 1.1 score (simplified)	No response (SD + PD)	82	56	Reference	–	–	–
	Response (CR + PR)	39	22	0.85 (0.51–1.40)	0.521	–	–
Post-treatment resectable status change (simplified)	Unchanged	96	60	Reference	–	–	–
	Downstage	20	15	1.66 (0.94–2.94)	0.081	–	–
	Upstage	5	3	1.20 (0.37–3.86)	0.760	–	–
Post-neoadjuvant serum CA 19-9 reduction from baseline	< 45% reduction	9	5	Reference	–	–	–
	45–85% reduction	41	26	1.05 (0.40–2.77)	0.914	–	–
	≥ 85% reduction	34	22	0.69 (0.26–1.83)	0.455	–	–
Surgical resection	Pancreatoduodenectomy	100	62	Reference	–	–	–
	Distal pancreatectomy	21	16	0.95 (0.55–1.66)	0.869	–	–
Positive margin on specimen	No	106	69	Reference	–	–	–
	Yes	15	9	1.40 (0.69–2.83)	0.345	–	–
College of American Pathologists treatment effect	Score 3	31	26	Reference	–	Reference	–
	Score 0–2	90	52	0.54 (0.34–0.88)	**0.013**	0.61 (0.36–1.02)	0.059
Histopathological tumor grade	Well-differentiated—Grade 1	19	12	Reference	–	–	–
	Moderately differentiated—Grade 2	53	31	1.09 (0.56–2.14)	0.797	–	–
	Poorly differentiated—Grade 3	32	22	1.25 (0.62–2.55)	0.532	–	–
Positive lymph nodes in specimen	No	64	35	Reference	–	Reference	–
	Yes	57	43	2.64 (1.66–4.20)	**< 0.001**	2.21 (1.35–3.62)	**0.002**

Bold text denotes significant *p* ≤ 0.05

*CI* confidence interval, *ECOG* Eastern Cooperative Oncology Group, *NCI* National Cancer Institute, *NCCN* National Comprehensive Cancer Network

## Discussion

In this contemporary, single-institution cohort of resectable and borderline-resectable pancreatic ductal adenocarcinoma (PDAC) treated with multiagent NAT, several trends among radiologic, biochemical, and histopathologic response metrics were observed: (1) radiographic response by RECIST was uncommon; (2) histopathologic regression after resection was prevalent and more closely associated with survival; and (3) isolated unresectable local progression was rare, although overall attrition during NAT affected a meaningful proportion of patients. Together, these findings highlight the importance of using multiple assessment modalities to evaluate neoadjuvant response and guide surgical decision-making in PDAC.

In this analysis, most patients had stable disease on restaging imaging, yet a substantial proportion showed meaningful histopathologic response after resection (CAP 0–2). This discrepancy is consistent with prior studies demonstrating limited correlation between radiologic and histopathologic response in PDAC treated with neoadjuvant therapy, reflecting the inherent limitations of RECIST in detecting therapy-induced microscopic stromal changes that may profoundly influence survival.^15,26^ More broadly, assessment of treatment response after neoadjuvant therapy for PDAC using cross-sectional imaging remains challenging, and expert radiologic reviews have emphasized that changes in tumor size alone on imaging may under-represent underlying biologic response.^27^ Clinically, the findings in this study support a treatment paradigm in which the absence of radiographic response on restaging imaging should not be equated with treatment failure in the absence of objective disease progression or clear anatomic unresectability. Moreover, isolated unresectable local progression after NAT—which could represent a missed opportunity for curative surgery—occurred infrequently in this analysis, with most cases accompanied by systemic progression, indicating aggressive tumor biology and the likely futility of resection from the outset. Prior studies report similar findings and suggest that, even when isolated unresectable local progression does occur, surgical resection with vascular reconstruction is often still feasible.^28^ However, it should be noted that the low rate of isolated unresectable local progression does not capture the full scope of treatment-related attrition; in this cohort, 27% of patients did not proceed to resection due to a combination of disease progression, treatment intolerance, and clinical deterioration (Supplementary Fig. 1). While these outcomes largely reflect systemic disease biology and patient fitness rather than a failure to preserve local operability, they represent a clinically meaningful risk of the neoadjuvant approach.

By contrast, biochemical response appeared to provide a stronger signal for treatment effect. In this cohort, serum CA 19-9 decline ≥ 85% was associated with a substantial survival benefit (median OS 56 *vs.* 36 months), although this association was ultimately shown not to be independently predictive of improved survival (OS HR 0.69, 95% CI 0.26–1.83; *p* = 0.46). Prior studies have similarly found that CA 19-9 response to neoadjuvant therapy is associated with R0 resection, histopathologic response, and survival.^20,29^ As such, serum CA 19-9 remains a valuable component of pre- and postoperative risk stratification for patients with PDAC, and institutional practices have more frequently begun using it as a criterion for altering neoadjuvant regimens when patients show suboptimal biochemical response.^30^ While optimal interpretation of CA 19-9 kinetics remains an ongoing area of investigation, this perioperative monitoring of serum CA 19-9 falls in line with the increased emphasis on demonstrating treatment effect prior to resection in PDAC.^29,30^

Finally, histopathologic response provided additional prognostic discrimination in the patients who underwent resection. While complete or near-complete pathologic regression is uncommon in PDAC, it is consistently associated with favorable outcomes.^31^ The results in this analysis fit within this framework: histologic regression captured treatment effect not seen on imaging, coinciding with improved long-term outcomes. This has practical implications for postoperative counseling and risk stratification, supporting tumor regression grading as a clinically meaningful endpoint that should be emphasized in ongoing or future clinical trials.

Importantly, the discordance across response domains reinforces that, in the absence of absolute contraindications to resection, operative decision-making following neoadjuvant therapy should not hinge on imaging response alone. The cohort in this analysis demonstrates that patients without radiographic response can still benefit from resection, and treatment effect may not be fully appreciated until histologic response is evaluated. Conversely, radiographic progression or rising CA 19-9 indicates aggressive disease biology and should prompt careful restaging and multidisciplinary discussion on the utility of surgical resection. Notably, in this cohort, disease progression during the neoadjuvant period was predominantly metastatic in nature. This aligns with established neoadjuvant paradigms in PDAC, where treatment is used both to improve the likelihood of margin-negative resection and to select patients with favorable tumor biology to minimize futile surgical intervention. Similarly, contemporary neoadjuvant approaches incorporating multiagent chemotherapy followed by chemoradiation in borderline-resectable disease underscore the need for endpoints beyond just decrease in tumor size.^32^

This study has limitations inherent to retrospective analyses, including potential selection bias due to inclusion of only patients considered for resection, heterogeneity in the timing of post-treatment restaging, and limited informativeness of CA 19-9 in Lewis antigen-negative patients. Nevertheless, the reported data support a multimodal response framework for PDAC after neoadjuvant therapy. Future work should focus on refining composite response criteria utilizing a combination of biochemical, imaging, performance-status, or novel precision medicine tools and validating them against recurrence and survival. Additionally, dedicated effort should be given to advancing imaging techniques that better distinguish viable tumor from treatment effect.

## Conclusions

This study provides a contemporary framework for understanding PDAC response markers in the era of modern systemic therapy, highlighting key insights: (1) response and downstaging can occur with NAT; (2) radiologic response after NAT does not always correlate with histopathologic response and thus should not preclude surgical decision-making; (3) imaging response to NAT does not necessarily predict long-term survival; and (4) isolated unresectable local progression on NAT is rare, though broader treatment-related attrition remains a consideration in neoadjuvant sequencing. Future studies should focus on integrating radiologic, pathologic, and molecular biomarkers to better define biologic response, refine patient selection for surgery, and improve prognostication following neoadjuvant therapy.

## Supplementary Information

Below is the link to the electronic supplementary material.

Supplementary file1 (DOCX 345 kb)
