# Supplementary material for: Assessing Response and Outcomes to Modern Multiagent Neoadjuvant Therapy for Pancreatic Ductal Adenocarcinoma
Source: Ann Surg Oncol. 2026 Jun 19;33(9):8448–58. doi: 10.1245/s10434-026-19990-7 (PMC13452754; doi:10.1245/s10434-026-19990-7)
Supplement: Supplementary file 1 [file 10434_2026_19990_MOESM1_ESM.docx]

**Supplementary Figure 1 –** Cohort definitions and patient attrition during neoadjuvant therapy and prior to surgical resection.

**Supplementary Figure 2** – Kaplan-Meier survival curves showcasing **(A)** progression-free survival (PFS) and **(B)** overall survival (OS) in patients with resectable or borderline-resectable PDAC, stratified by combined nonresponders (RECIST + biochemical criteria) vs. patients with any response.

**Supplementary Figure 3** – Kaplan-Meier survival curves showcasing **(A)** progression-free survival (PFS) and **(B)** overall survival (OS) in patients with resectable or borderline-resectable PDAC, stratified by alternative CAP score response criteria.
